# MLGA: a cost-effective approach to the diagnosis of gene deletions in eye development anomalies

**Published:** 2009-07-28

**Authors:** Alexander W. Wyatt, Nicola Ragge

**Affiliations:** 1 Department of Physiology, Anatomy and Genetics, University of Oxford, Oxford, UK; 2 Moorfields Eye Hospital, London, UK; 3 Department of Ophthalmology, Birmingham Children’s Hospital, Birmingham, UK

## Abstract

Whole gene deletions or duplications are an important cause of genetic disease and phenotypic variation. Targeted techniques for the routine testing of gross rearrangements have become essential tools for diagnostic researchers with the search for the most cost-effective and efficient tool assuming high priority. We used the new selector technique, MLGA (multiplex ligation-dependent genome amplification), to confirm deletions in two genes, *SOX2* (SRY [sex determining region Y]) box 2) and *OTX2* (orthodenticle homeobox 2), in individuals with developmental eye disease. We conclude that MLGA has the potential to be a useful technique in diagnostic research for the identification of deletions or duplications of known genes due to its speed and relatively low cost.

## Introduction

Copy number variation (CNV) including submicroscopic deletions, insertions, and duplications of DNA sequences is becoming increasingly recognized as both an important source of genome variation and a common cause of genetic disease [1-4]. Over recent years, a variety of techniques have been designed to identify CNVs. Global microarray-based approaches such as array-CGH (comparative genomic hybridization) are widely used to detect CNVs on a genome wide scale, but costs are still relatively high compared to targeted approaches. Simple targeted approaches such qPCR (quantitative polymerase chain reaction) and FISH (fluorescence in situ hybridization) are well established but restrictive due to their inability to be multiplexed and their labor-intensive nature. Techniques without these shortcomings such as multiplexed targeted approaches including MAPH (multiplex amplifiable probe hybridization) [5], QMPSF (quantitative multiplex polymerase chain reaction of short fluorescent fragments) [6], PRT (paralogue ratio test) [7], and MLPA (multiplex ligation-dependent probe amplification), which is perhaps the most commonly used [8], have become essential tools for the routine detection of both large deletions/duplications and exonic/single gene CNVs. Indeed, we have recently used MLPA to detect deletions in *SOX2* (SRY (sex determining region Y)-box 2), *OTX2* (orthodenticle homeobox 2) and *BMP4* (bone morphogenetic protein 4), genes involved in severe eye abnormalities [9-11]. However, despite its simple and robust nature, MLPA is still relatively expensive and suggests the need for a more cost-effective method of detecting CNVs.

We evaluated a new selector technique, multiplex ligation-dependent genome amplification (MLGA), which has the potential to significantly reduce cost and turnaround time. In MLGA, size-coded genomic DNA fragments are circularized and undergo multiplex amplification using universal primers [12]. The time taken per MLGA assay is less than 5 h with only three brief steps required before PCR amplification. Only one probe is required per target (as opposed to two in MLPA), and the running costs are less than a fifth of the cost of MLPA. However, no follow-up publications to the initial report have emerged, and despite its potential, MLGA has yet to be validated in a human diagnostic setting. We evaluated the use of MLGA to detect known deletions in five patients with severe developmental eye anomalies.

## Methods

Patients with developmental eye anomalies (anophthalmia and/or microphthalmia) were recruited to this study as part of a national study based at Moorfields Eye Hospital (London, UK) and Birmingham Children’s Hospital (Birmingham, UK). Written informed consent was obtained from all patients in accordance with the ethics approval, which was obtained for the study from Cambridgeshire Research Ethics Committee 1 04/Q0104/129 (Cambridge, UK). Genomic DNA was extracted from whole blood using standard protocols.

MLGA was performed according to the protocol described by Isaksson et al. [12], using their oligonucleotide sequences for the vector and universal primers. In essence, genomic DNA was digested by the restriction enzyme, MnlI. Restricted fragments were then circularized in a ligation reaction with the selector probes (see below) and vector oligonucleotide. The vector hybridizes to the central part of the selector probe, creating a HindIII recognition site and universal primer binding sites. Following the exonuclease I treatment to eliminate non-circularized DNA, PCR with the universal primer pair amplified the selected targets. The restriction enzyme, HindIII, was used during the PCR reaction to create a linear template for amplification. PCR products were run on an ABI Prism® 3130 (Applied Biosystems, Foster City, CA) and analyzed using ABI GeneMapper® (Applied Biosystems). All probes were initially tested in a cohort of 48 DNA samples without any gross rearrangements at the interrogated loci with probes for the X and Y chromosome (*AR* [androgen receptor] and *SRY* [sex determining region Y], respectively) to allow for experimental validation in the form of sex confirmation. The five positive cases with known deletions (Table 1) were tested with five normal control samples. Each assay was conducted in duplicate. Normalization of each sample trace was performed by dividing each peak area by the sum of its nearest two autosomal peak areas. Copy number ratio between samples was determined by dividing each normalized peak area by its mean across normal controls (as described [12]).

**Table 1:** Details of the known deletions in cases 1–5.

**Case**	**Previously detected by**	**Extent of deletion**	
		**Method**	**Region**
1	MLPA [11]	Oligo array-CGH	chr14:56,268,037–57,541,514
2	MLPA [9]	Deletion cytogenetically visible	46XX del(14)(q22.3q23.2)
3	MLPA [11]	Oligo array-CGH	chr14:53,758,044–56,834,649
4	MLPA [10]		Unknown
5	MLPA [10]		Unknown

Table showing method(s) of detection and extent of the gene deletions where known in cases 1-5.

Selector probe design was performed as described by Isaksson et al. [12] with two minor modifications, which is described in the following. First, reference sequences were obtained from the UCSC genome browser [13]: *OTX2* (GenBank accession number NM_172337.1), *SOX2OT* (*SOX2* overlapping transcript) (GenBank RNA accession number BC041898.2), and autosomal control gene, *NPM1 *(nucleolar phosphoprotein B23, numatrin) (GenBank accession number NM_002520.5). Second, newly designed selector probe and fragment sequences were uploaded to the BLAT alignment tool [14] to check for single nucleotide polymorphisms (SNPs) within probe hybridization sequences and length polymorphisms in the fragments. The overlapping transcript of *SOX2* (*SOX2OT*) was selected for interrogation due to the relative ease of optimization compared with the GC-rich *SOX2* itself. In addition to the control probe, *NPM1*, mentioned above, selector probes for control genes, *MADH4* (mothers against decapentaplegic homolog 4), *SRY*, and *AR*, were used as designed by Isaksson et al. [12]. Oligonucleotide sequences are shown in Table 2.

**Table 2:** List of oligonucleotides used in the MLGA protocol.

**Oligonucleotide**	**Sequence (5′→3′)**
SOT2OT	CTCTTAGCTTGGTTTCCTCCAGTCCACGATAACGGTAGAAAGCTTTGCTAACGGTCGAGTTTTGAAAACAGACGATAGAAGTCT
OTX2	CCAATTCACTCCCCCCTCTAGCACACGATAACGGTAGAAAGCTTTGCTAACGGTCGAGCAGAATGGAGGTCAAAACAAAGTGA
NPM1	GTCCCGCCTCCGCGCGACGATAACGGTAGAAAGCTTTGCTAACGGTCGAGCTCATGTCCATGTCCATCGAATCTT
Vector	CTCGACCGTTAGCAAAGCTTTCTACCGTTATCGT
Forward primer	AGCTTTGCTAACGGTCGAG
Reverse primer	AGCTTTCTACCGTTATCGT

Table showing oligonucleotide sequences for additional selector probes (*SOX2OT*, *OTX2* and *NPM1*) not described in the original MLGA report by Isakson et al. [12], the vector used during circularisation and the universal primers used for multiplex amplification.

## Results

**Figure 1:**
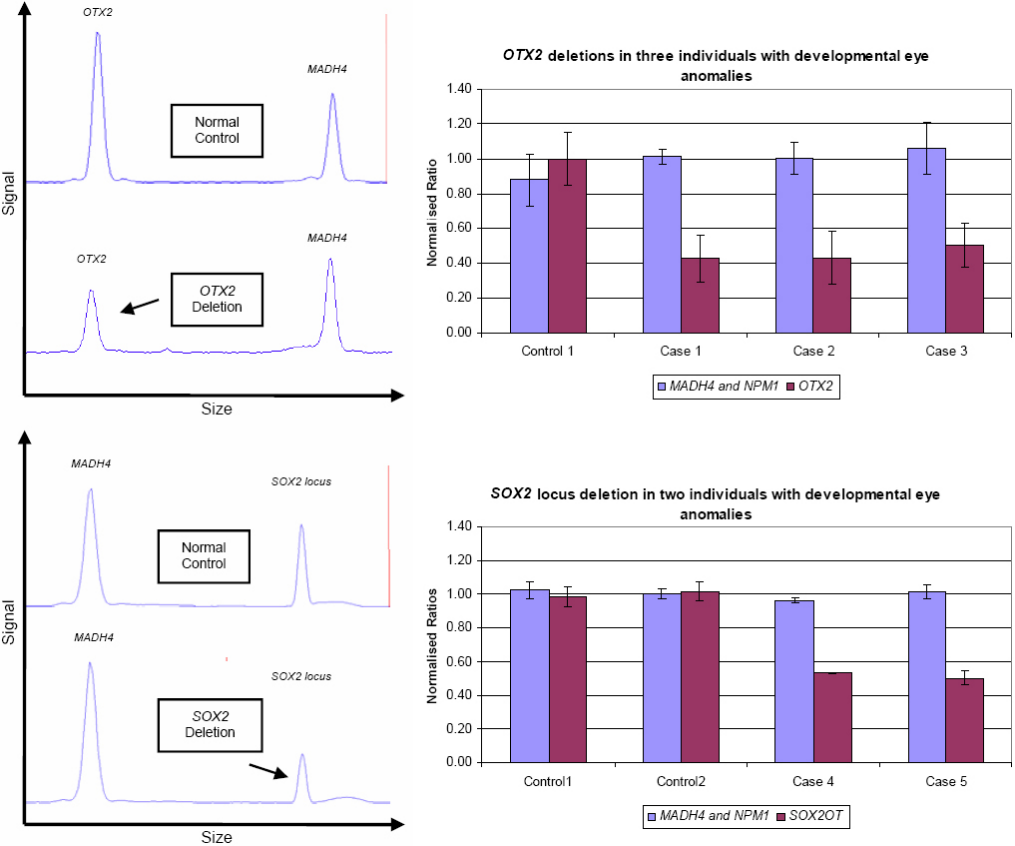
The use of MLGA to detect *OTX2* and *SOX2* deletions in cases 1-5. Left: GeneMapper® traces demonstrate the reduced signal from an *OTX2* deletion (upper) and a *SOX2* locus deletion (lower). Right: Bar graphs of normalized peak area ratios show reduced copy numbers of *OTX2* (upper) and *SOX2* locus (lower) in individuals with deletions when compared to normal controls. A normalized ratio of 0.5 indicates a deletion of one copy of the targeted loci while a ratio of 1 indicates a normal copy number.

The ABI GeneMapper® traces demonstrated a proportional reduction in the peak area of the *OTX2* fragment in all three individuals (cases 1–3) with previously identified *OTX2* deletions and a proportional reduction in the peak area of the *SOX2* locus fragment in individuals (cases 4 and 5) with previously identified deletions of the entire *SOX2* locus (Figure 1 for traces and graphs of *OTX2* and *SOX2* locus deletions). Each result was confirmed in a second assay.

## Discussion

Our results demonstrate that MLGA can identify whole gene deletions in a human diagnostic research setting as exemplified by the diagnosis of human *OTX2* and *SOX2* deletions in developmental eye anomalies. For MLGA to become widely used in a diagnostic service setting, further detailed validation work is required to prove reproducibility in a large number of positive cases and controls. However, the low running costs along with the ease and speed of the assay would be particularly attractive to human genetic diagnostic laboratories requiring regular CNV screens of known disease loci. By amplifying genomic DNA rather than probe molecules, MLGA assays offer the potential to screen at least 50 loci in one assay by using several vectors and multiple labels on the universal primers. Although the routinely used technique, MLPA, is able to analyze this number of loci, MLGA has the potential to do this faster and more economically. Furthermore, MLGA provides great flexibility in the choice of target loci through the library of restriction enzymes available to create fragments for subsequent circularization and amplification.

MLGA still requires further validation when multiplexing up to 40-50 loci for it to be deemed as useful as other multiplex targeted approaches (including MLPA) in CNV detection. Moreover, significantly multiplexed assays may require more stringent selector probe design criteria and a more thorough initial optimization of reaction conditions than MLPA since genomic DNA rather than designed probe sequences are amplified. Aside from repetitive elements, most of the genome is accessible to MLGA with only high GC content (>60%) known to affect probe success rates [12].

One significant advantage that MLGA does possess but was not noted in the initial report is the capacity to provide data on insertion-deletion polymorphisms or mutations with a target sequence of interest. For example, a two base pair deletion within a target sequence will give a shifted peak on the GeneMapper® trace and thus may indicate an avenue for further investigation, such as sequencing. This potential may prove valuable to a researcher screening for mutations as well as deletions, providing a useful ‘pre-screen’ to detect intragenic deletions or insertions.
